# SMaTE: A Segment-Level Feature Mixing and Temporal Encoding Framework for Facial Expression Recognition

**DOI:** 10.3390/s22155753

**Published:** 2022-08-01

**Authors:** Nayeon Kim, Sukhee Cho, Byungjun Bae

**Affiliations:** 1 Communication and Media Engineering, University of Science and Technology, 217, Gajeong-ro, Yuseong-gu, Daejeon 34113, Korea; boboss@etri.re.kr; 2 Electronics and Telecommunications Research Institute, 218, Gajeong-ro, Yuseong-gu, Daejeon 34129, Korea; shee@etri.re.kr

**Keywords:** video, deep learning, facial expression recognition (FER), transformer

## Abstract

Despite advanced machine learning methods, the implementation of emotion recognition systems based on real-world video content remains challenging. Videos may contain data such as images, audio, and text. However, the application of multimodal models using two or more types of data to real-world video media (CCTV, illegally filmed content, etc.) lacking sound or subtitles is difficult. Although facial expressions in image sequences can be utilized in emotion recognition, the diverse identities of individuals in real-world content limits computational models of relationships between facial expressions. This study proposed a transformation model which employed a video vision transformer to focus on facial expression sequences in videos. It effectively understood and extracted facial expression information from the identities of individuals, instead of fusing multimodal models. The design entailed capture of higher-quality facial expression information through mixed-token embedding facial expression sequences augmented via various methods into a single data representation, and comprised two modules: spatial and temporal encoders. Further, temporal position embedding, focusing on relationships between video frames, was proposed and subsequently applied to the temporal encoder module. The performance of the proposed algorithm was compared with that of conventional methods on two emotion recognition datasets of video content, with results demonstrating its superiority.

## 1. Introduction

Emotions are unintended responses that occur automatically when humans are stimulated by stimuli such as an event or interaction. Emotion recognition is the classification of the emotions of a person that appear in various multimedia content such as images, videos, speech, and text, or in the context of such multimedia content [1]. It provides an adaptive approach that aids in better understanding the events or interactions in a complex and changing world through the detection of psychological changes in people.

Facial expression shown in the images of people is among the key methods for recognizing emotions. These visual responses are extended to application programs to provide important clues for more accurate emotional interactions in human–computer interactions (HCI). Further, studies on recognizing emotions using speech, which is the foundation of human communication, have also been conducted for a long period. Facial expression recognition (FER) [2,3,4,5,6,7,8] and speech emotion recognition (SER) [9,10,11,12,13] have existed for over 20 years. However, with recent advances in deep neural networks, FER and SER have once again gained attention.

In recent years, powerful and reusable deep learning technologies based on large-scale training data and the power of high-performance computation have been suggested as alternatives to existing technologies in almost all fields, including emotion recognition. As convolutional neural networks (CNNs) have outperformed humans in various pattern recognition tasks by learning advanced features in image big data, researchers have begun reviewing CNN extensively from various perspectives [14,15,16,17,18]. Recurrent neural networks (RNN) can effectively classify speech and text owing to their ability to handle sequential sequence data of a long distance [19,20,21]. Consequently, many researchers have adopted RNN and CNN models to achieve excellent results in application programs for FER [8,22,23,24,25,26] and SER [27,28]. In addition, emotion recognition multimedia application programs based on these research results have been utilized as one of the major or minor functions throughout society, including robots, entertainment, social media industry, healthcare, and welfare.

However, there has been a recent emergence of a massive amount of video data in the visual world. Videos are being created continuously at places such as public facilities to everyday lives through CCTVs, home cameras, online lectures, social media, and medical video. Therefore, video-based emotion recognition has become a necessity. Many researchers have begun focusing on fusion networks based on multimodal data to detect more complex structures and features by exploiting the features of videos [29,30], which contain both images and speech. Further, the DeepBlueAI research team [31], which achieved third place in the eighth Emotion Recognition in the Wild (EmotiW) 2020 challenge [32], fused up to 14 models. In addition, the SituTech research team [33] achieved first place with a hybrid network wherein seven types of data streams were fused.

Based on several empirical evidence, the multimodal approach, which fuses various types of data, has been confirmed to outperform existing single method models. However, the applicability of this approach in the real-world application domains needs to be discussed. In computer-aided diagnosis systems, image analysis and processing are essential parts of interpreting and detecting most diseases from medical video. From a crime prevention perspective, emotions and situations must be captured through facial expressions rather than voice to determine the situation for emotion recognition that can be used to prevent crimes through CCTV, arrest a suspect or a criminal, or detect digital sex crimes through social media. In addition, another problem from the perspective of an individual’s identity is encountered in the real world. It stems from the fact that facial expressions may appear differently according to various races, cultures, looks, gender, and age. Furthermore, the detection of subtle facial motions and head movements within natural expressions is challenging. These problems can only be solved through research on recognizing facial expressions from image sequences that have been extracted from a video, excluding speech, which is one form of data among components of a video. However, even the team [34] that accomplished the best result at EmotiW challenge [35] using the Acted Facial Expressions in The Wild (AFEW) dataset [36,37] (widely known as a wild video emotion dataset) achieved low-quality results when using only image sequences. They achieved a best performance of 49.30% using a multimodal fusion model and only 39.69% with the model employing only facial images [38]. This result indicates the prevalent low accuracy of an individual performance on image sequence.

An image sequence has both spatial and temporal information. Therefore, the CNN–RNN architecture [39], which employs a combination of CNN and RNN, was used in many of the previous studies. However, CNN is a process of finding a type of template filter that operates universally on image data. In addition, covering all identities of an individual is challenging because parameters such as the input and weights are fixed after training completion. Moreover, RNN renders the performing of data parallelism challenging because it can learn only through sequential receival of sequence data as the input.

Therefore, this study proposes a segment-level feature mixing and temporal encoding (SMaTE) framework, in an attempt to tackle these problems. First, the SMaTE framework was designed based on a reusable vision transformer (ViT) [40] to facilitate easy application to real-world facial expression recognition application programs. The proposed model comprised two separate transformer encoders to enable the learning of spatial and temporal information. As this model first extracted the spatial information and then modeled the interactions between temporal indices, it is similar to the CNN–RNN architecture that was widely used in previous studies. However, the proposed model can process image sequence data in parallel because it is based on the transformer [41] model. Moreover, it was designed to facilitate augmentation of image sequences using several effective methods and subsequently fuse them within the FER transformer model. Furthermore, the proposed method applied the position embedding technique, which primarily focused on temporal information compared to spatial information, in a temporal encoder that modeled the temporal interactions.

The primary contributions of this study are as follows:

1. A new framework referred to as SMaTE is proposed for facial expression recognition based on the video vision transformer model.
2. Data augmentation and feature extraction were performed, with the aim being for the model to learn useful representations of FER more effectively. Various data enhancements were decomposed into patch units and converted into token sequence through linear projection. Subsequently, these were randomly aggregated into one token architecture and thus improved the modeling of FER.
3. Temporal position encoding is proposed for FER instead of the learned position embedding used in the existing work [42]. This encourages increase in the interaction between data and position encoding in temporal transformer encoder. This study shows that the proposed encoding methods outperform existing position embeddings on the Extended Cohn–Kanade (CK+) and AFEW.
4. The proposed framework was demonstrated to be the best choice for improving FER performance with only a few adjustments of pure-transformer architectures [42] through ablation analysis of the position encodings, data augmentation and feature extraction methods, tokenization strategies, and a model architecture.

The Extended Cohn–Kanade (CK+) dataset [43,44] is a laboratory-based dataset, whereas the AFEW dataset is a wild environment dataset for video-based FER. The experiments conducted using two types of facial expression recognition datasets demonstrated that the SMaTE model can improve the recognition rate of facial expression sequences in both laboratory and wild environments. It also showed that the SMaTE model can achieve better results than the existing methods.

The remainder of this paper is organized as follows: Section 2 introduces the related motivations and tasks, and Section 3 describes the SMaTE model in detail. Further, Section 4 introduces the details of the experiment and evaluates the performance. Finally, Section 5 presents the conclusions.

## 2. Related Works

Researchers have proposed effective previous methods such as data preprocessing and feature extraction to solve classification problems for images that are difficult to interpret, such as vague boundaries in skin lesions detection and appearance bias in FER. Data preprocessing removes unwanted parts of the image that can be misinterpreted to classify the target. Feature extraction extracts distinct features from images that play an important role in classification tasks. Handcrafted features manually design and extract elements of the image that are relevant to the target through prior knowledge [45]. On the other hand, non-handcrafted features were optimized features extracted through deep learning and showed excellent detection accuracy in various fields [46].

In previous FER works, to address the problems discussed, fusion models such as multiple modalities that combine various data (e.g., speech, brain, and peripheral signals) [31,33,34,38] and multi-task learning that utilizes commonalities and differences across tasks at the same time [47,48,49,50] have been studied. Reference [49] proposed a fusion model that combined the two tasks to determine the optimized final loss from individual identity-based face recognition and FER architecture. Further, references [51,52] proposed fusion networks wherein features of face and semantic context were extracted from input images and combined.

Deep neural networks, combining CNN and RNN, have mostly been used to classify image sequences. CNNs [14,15,16,17,18] can effectively model the spatial relations of image components, whereas RNNs [19,20] are advantageous in learning relationships within a sequence [53,54,55,56]. These models are used via the combination of CNN as an encoder and RNN as a decoder to extract features of frames and spatiotemporal features.

CNNs can learn and share several similar patterns by extracting features from various portions while multiple convolution filters slice the image area based on the common assumption of translation equivariance. These filters can efficiently learn the model by sharing parameters for each target class [57,58].

While multiple convolution filters slice the image area based on the property of translation equivariance, CNNs can learn several similar patterns through the extraction of features from various portions. These filters can efficiently learn the model by sharing parameters for each target class. Therefore, a CNN is translation-invariant and the output does not change regardless of changes in position of the target object (e.g., facial expression) or variation in its appearance in the input image. CNN exhibits superior performance in image classification because it has correct assumptions regarding the following nature of images [59]: locality assumption, which tends to have a stronger relationship with adjacent pixels, and stationarity assumption, where the same patterns are repeated.

Nevertheless, appearance biases still influence prediction. Further, performance decline due to individual characteristics (appearance, culture, gender, age, etc.) is a problem that often occurs in classification using face images such as facial recognition and facial expression recognition. Classification of even a well-generalized FER CNN model can be challenging if facial expression intensity or patterns differ due to its appearance bias across race and region, and response bias across cultures [60,61,62]. A person whose neutral expression is similar to that of a smiling face or angry face can cause a CNN-based FER model to yield incorrect results. This problem is particularly pronounced in video FER, where movement and facial expression intensity are not constant.

**Figure 1:**
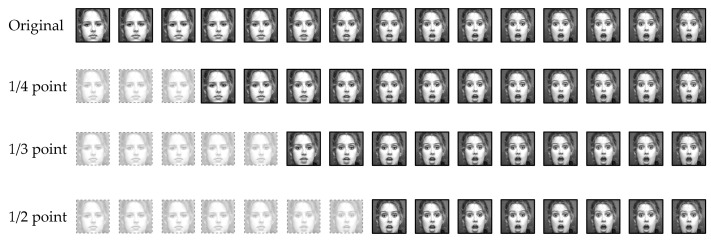
Transformation of the start point of sequence on the Extended Cohn–Kanade (CK+).

This study transformed the CK+ dataset, traditionally used for FER, to measure accuracy according to human appearance bias. The CK+ dataset is a dataset representing a sequence of images from Neutral to the maximum intensity of seven categorical emotions for each subject. As shown in Figure 1, image sequences that changed the starting frame to $\frac{1}{4}$, $\frac{1}{3}$, and $\frac{1}{2}$ points were added for the training set. Thus, the impact of the appearance bias was evaluated by training data that changed the unique neutral expression of a person.

**Figure 2:**
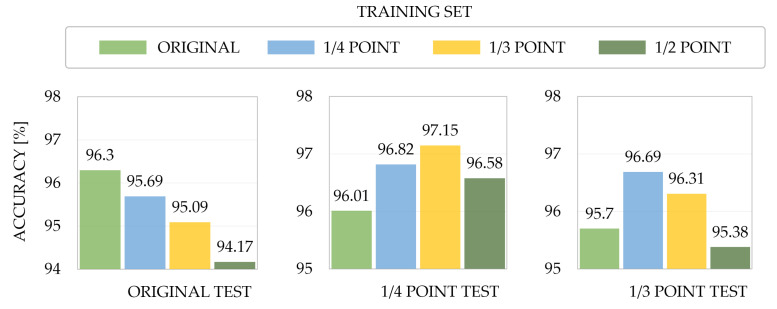
Ten-fold cross-validation accuracy for training sets corresponding to each transformation method in the three test sets (original, 1/4 point, 1/3 point).

To perform the experiments, a face-related multi-task model based on EfficientNet [63] was used. It has an acceptable accuracy while having a relatively small size. Figure 2 shows the results following 10-fold validation under different neutral expression data. The model, which learned with $\frac{1}{4}$ point and $\frac{1}{3}$ point data, exhibited higher performance in all test sets, including appearance biases, than the model that learned only with the original data. Thus, based on the above observations, this study emphasizes the need for a robust FER framework including data preprocessing, combination of handcrafted features and deep learning-based features, and a transformer-based model to consider appearance biases with diverse backgrounds.

## 3. Proposed Methods

This section describes the SMaTE framework, which aims at (i) flexible learning to appearance bias and (ii) increased temporal interaction between the embedding token and position encoding, based on video vision transformer (ViViT). First, the data augmentation and feature extraction technique are described, along with the data preprocessing methods for facial expression recognition. Subsequently, the three main components in SMaTE framework, mixed-token embeddings in Section 3.2.2, temporal positional encoding in Section 3.2.3, and transformer encoder in Section 3.2, are discussed.

### 3.1. Preprocessing for FER

**Figure 3:**
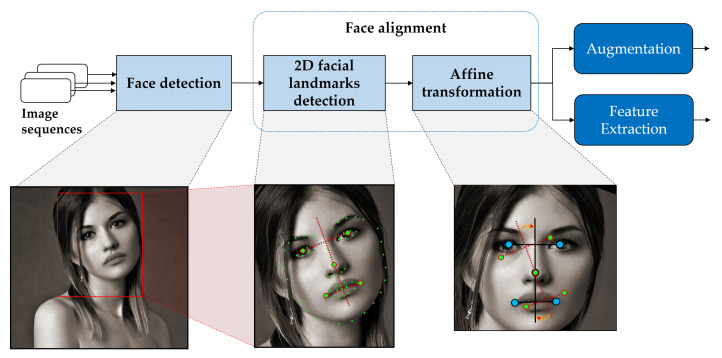
Overall procedure for face preprocessing (face detection and face alignment), data augmentation, and feature extraction.

In general, face image tasks share a common data processing that obtains uniform shape and normalized data and prevents learning unnecessary representation. The overall steps for FER preprocessing are shown in Figure 3. Face preprocessing, which entails face detection and face alignment, was performed to extract a facial expression image sequence from the video. Thereafter, the preprocessed face sequences were augmented followed by extraction of features to address training data scarcity and mitigate appearance bias.

#### 3.1.1. Face Preprocessing

In the face detection process, the face sequence is generated through the detection and cropping from each image containing the face in the video. Face alignment can efficiently handle pose, thereby improving the performance of demanding FER tasks. The face preprocessing method comprised three steps: face detection, landmark detection, and affine transformation in face alignment.

**Face detection.** Recent research results of deep-learning-based face detection have already confirmed its effectiveness. Most face detection models receive images as input and return a bounding box and a confidence score for each face. This study used the Light and Fast Face Detector (LFFD) [64], which is a face detector that balances accuracy and latency and focuses on fast processing data such as large-scale images/video. It yielded a result of 89.3% on the WIDER FACE [65] benchmark dataset. Further, it can run at 131.4 fps faster than other models with a minimum and maximum of 12.81 and 81.11 fps, respectively, in the same experimental environment, with a difference of only 2%.

**Landmark detection**. Face alignment ensures correspondence to the same location of the face regions detected from different face images for a better face understanding. Landmark localization is a process of detecting facial landmark such as eyes, nose, eyebrows, and jawline in the face as part of the face alignment task. The detected landmark points are used as a reference for aligning the data. The landmark detector estimates landmark localization by modeling the geometric structure with the already detected face region as an input. This study used a PFL [66] that returned a 68-point landmark position at an average of 100 fps.

**Affine transformation**. Following the identification of a reference point through landmark detection in face alignment, each pixel is aligned in the points through transformation. This study employed affine transform, which preserves lines and parallelism and allows in-plane of rotation, translation, scale transformation, and shear.

#### 3.1.2. Data Augmentation

**Figure 4:**
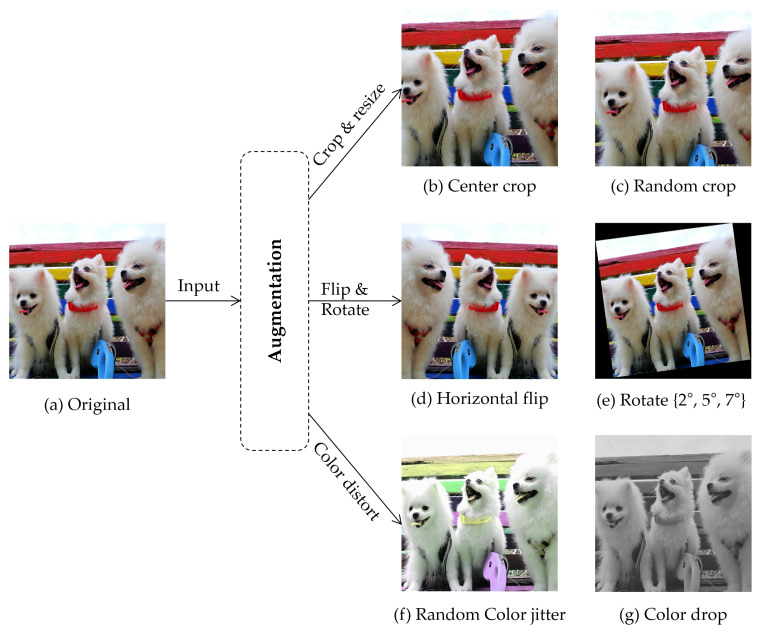
Examples of augmented data. All augmentation methods are only applicable to training of the model, except for cropping and resizing, which are used for testing. Photo by Pomfam20 licensed under CC BY 4.0.

Compared to CNN, ViT recently achieved more high scores in image classification; however, it has weak inductive biases, such as locality that allows CNN to reach high performance with small data. Thus, ViT requires large-scale data and its performance varies depending on the number of samples. Previous studies have shown that expanding the data space reduces overfitting and provides improvements in performance [67]. Data augmentation is an essential method for learning induction bias within the data by increasing the amount of labeled data in an insufficient dataset. This study utilized several augmentations, such as transformation in geometry, pixels, and color distort (drop and jitter), described in [68], as shown in Figure 4.

Geometric transformation comprises cropping, resizing, rotation, and flipping. Cropping is employed to obtain subimages of central or random locations from the original image. Thereafter, resizing is applied to maintain height and width dimensions. In facial expression recognition tasks, appropriate reduction thresholds for cropping should be selected to maintain the face shape and preserve a label. In flipping, only the horizontal flip is used. Further, rotation is performed by rotating the image to the right or left relative to the axis. To achieve facial expression-preserving transformation, the degree of rotation parameter is limited to $\left\{2^{\circ },5^{\circ },7^{\circ }\right\}$ [69].

Color transformation is not a label-preserving transformation in applications where color information is important; however, for FER tasks, it aids in analysis of spatial structures by eliminating color bias in training sets. This study augmented the data using color jitter techniques that simply converted RGB matrices to a single grayscale image, or randomly changed lightness, hue, and saturation.

#### 3.1.3. Feature Extraction

**Figure 5:**
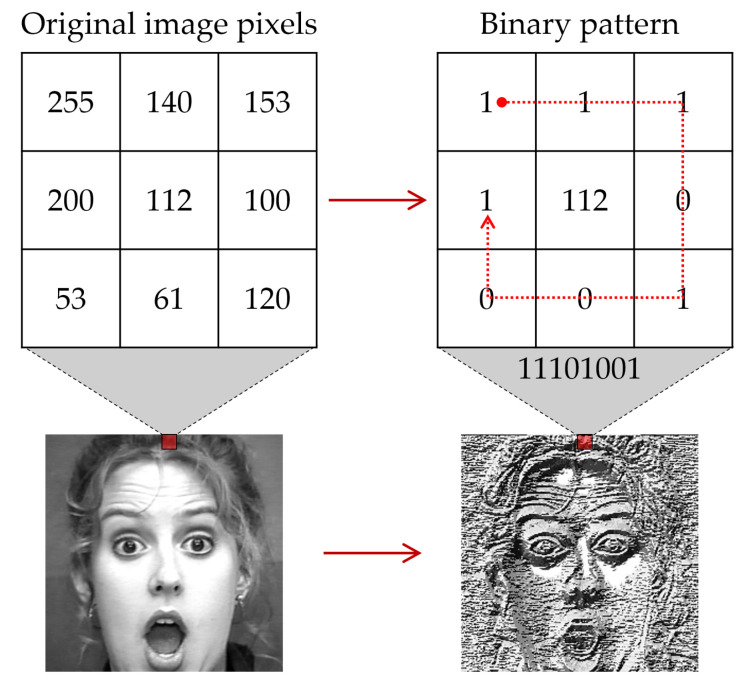
Example of face feature extraction method based on local binary pattern (LBP).

A local binary pattern (LBP), which is a simple and effective texture description operator, was applied as a feature extraction method for converting data into a high-level representation. LBP is a simple grayscale operator that describes the local spatial pattern. In face-related tasks, LBP is a robust feature that emphasizes the texture of local information of the human face, such as facial boundaries and muscle movements, which are key elements of FER, regardless of brightness changes. LBP was first applied in [70] and has since been used in facial recognition [71], facial expression recognition [72], and more. As illustrated in Figure 5, each neighbor, based on the selected central pixel $\left(x_{c},y_{x}\right)$ in 3×3 windows, is assigned a binary label by specifying a threshold. The feature of LBP is expressed as follows:

(1) $$LBP_{P,R}=\sum_{p=0}^{P-1}2^{p}\cdot S\left(g_{p}-g_{c}\right)$$

(2) $$S\left(g_{p}-g_{c}\right)=\left\{\begin{array}{c} 1,\;g_{p}-g_{c}\geq 0\\ 0,\;g_{p}-g_{c}<0 \end{array}\right.$$

where $g_{c}$ represents the value of the center pixel, and $g_{p}$ represents a neighborhood. All pixels are labeled with *S*, a threshold function. Further, depending on *S*, the difference between $p_{c}$ and $p_{n}$, that is, 1, was assigned to the pixel if it was greater than or equal to zero, and 0 if it was less than zero.

### 3.2. Model for Facial Expression Recognition

**Figure 6:**
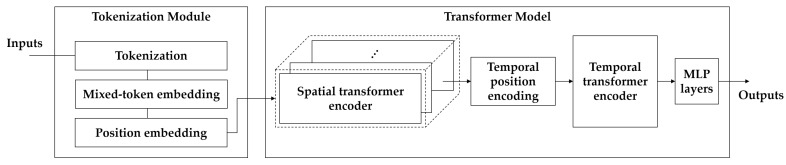
The proposed framework segment-level feature mixing and temporal encoding (SMaTE) comprises three main steps: (1) tokenization and token mixing for video sequences; (2) position embedding and position encoding for temporal transformer encoder; (3) transformer model for emotion classification based on video vision transformer (ViViT).

First, ViT [40] and video vision transformer (ViViT) [42] are briefly described in Section 3.2.1. The proposed framework is based on these two concepts. Thereafter, as illustrated in Figure 6, through the explanation of the manner in which tokens are extracted from the video in Section 3.2.2, the temporal position encoding method is proposed in Section 3.2.3. Finally, the backbone architecture is described in Section 3.2.4.

#### 3.2.1. Overview of Vision Transformer

This section introduces the ViT (deep learning model designed to learn images only with an attention mechanism) and ViViT (variant of ViT) as the baseline models. Transformers have been widely applied to NLP; however, recently the proposed ViT also reached the SOTA in large-scale image classification performance, and showed high-score performance in the various fields of computer vision. Subsequently, architectures based on transformers have been extensively studied in vision tasks, with ViViT being a variant of ViT specialized in video work. Standard self-attention operation underlying all transformer models is defined as follows:

(3) $$\mathrm{Attention}\left(Q,K,V\right)=\mathrm{SA}\left(z\right)=softmax\left(\frac{QK^{T}}{\sqrt{D}}\right)V$$

The similarity between query $Q=zw_{q}$ and key $K=zw_{k}$ in the input sequence $z_{i}=\left[z_{i}^{1},\;...,\;z_{i}^{n+1}\right]$ of the *i*-th transformer layer *ℓ*, can be calculated. Then, a weighted sum for all values $V=zw_{v}$ is computed (Equation (3)).

**Figure 7:**
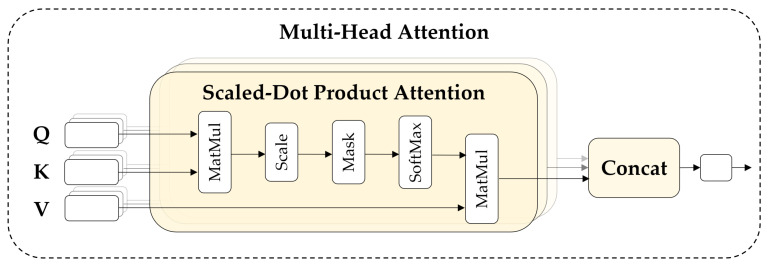
Structure of the multi-head attention mechanism.

As shown in Figure 7, multi-head self-attention (MSA) comprises multiple heads, representing the attention operation of different locations. Moreover, it allows the model to learn different representation information in different locations in parallel on the input token sequence *z* according to pure-transformer [41]. The number of heads is *k*, and $D_{h}$ is set to D/k. In the transformer, each layer *ℓ* of the encoder comprises an MSA, a layer normalization (LN), and a multi-layer perception (MLP) block, including two layers with a GELU nonlinearity [73] as follows:

(4) $$y^{\ell }=\mathrm{MSA}\left(\mathrm{LN}\left(z^{\ell }\right)\right)+z^{\ell }$$

(5) $$z^{\ell +1}=\mathrm{MLP}\left(\mathrm{LN}\left(y^{\ell }\right)\right)+y^{\ell }.$$

#### 3.2.2. Token Embeddings

**Tokenization**. ViT [40] performs a process referred to as patch embedding, wherein two-dimensional images are sequentially arranged and divided into N non-overlapping patches, because it directly applies a pure-transformer model that receives a 1D sequence as an input. In a similar manner, ViViT performs token embeddings that process n-dimensional videos $V\;\in R^{T\times H\times W\times C}$ to 1D input sequences.

**Figure 8:**
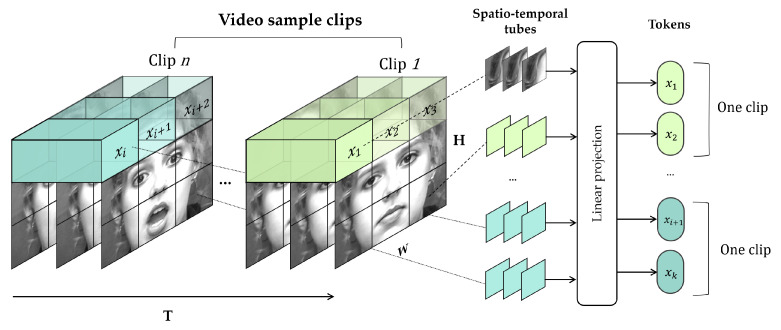
Tokenization using tubelet embedding method.

ViViT [42] uses two embedding methods to extend the patch embedding of ViT to video sequences: uniform frame sampling and tubelet embedding. Uniform embedding is a simple method for extending non-overlapping image patches of $n_{h}\times n_{w}$ to a total $n_{t}\times n_{h}\times n_{w}$ token over the time of the video clip, whereas tubelet embedding, described in Figure 8, extracts non-overlapping spatiotemporal tubelets that fuse spatiotemporal information from the image sequence.

Tokens are generated via linear projection of image patches (from uniform frame sampling) or spatiotemporal tubes (from tubelet embedding) extracted from a video, such as (6). Linear projection is a 2D convolution for uniform frame sampling and is similar to 3D convolution for tubelet frame sampling. $z_{data}$ is an embedding token obtained via linear projection of *k* extracted tubes, where data implies original data or augmented data.

(6) $$z_{data}=\left[Ex_{1},Ex_{2},\ldots ,Ex_{k}\right]$$

**Mixed-token embedding**. Mixed-token embedding was proposed to mix data randomly in latent space. As defined in Equation (7), a method is a notion of data augmentation that results in robust representations of facial expressions rather than individual identities. The embedding token and the mixed-token embedding are defined as follows.

To focus on representations of facial expressions rather than individual identities, the mixed-token embedding method was based on an interpolable latent space as follows (7). Subsets were randomly sampled from pairs of training token sequences $z_{origin}$ and augmented token sequences $z_{aug}$ via a random variable **X** that assigned a real number on a finite sample space 0,1. Thereafter, the sampled subsets, $z_{origin}$ and $z_{aug_{i}}$, were aggregated and represented as a 1D token sequence $z_{mix}$. Here, $z_{mix}$ is defined as original data token or data with augmented data mixed according to X. Each token, $z_{origin}$ and $z_{aug}$, was derived from the same video; thus, the same shape $R^{n_{t}\times n_{h}\times n_{w}\times d}$ facilitated the direct sum.

(7) $$z_{mix}=\left\{\begin{array}{cc} z_{origin}\; & \;\;\;\mathrm{if}\;X=0\\ \frac{1}{N+1}\left(z_{origin}+\overset{N}{\underset{n}{⨁}}z_{aug_{i}}\right)\; & \;\;\;\mathrm{if}\;X=1,n\in \left\{1..N\right\} \end{array}\right.$$

**Figure 9:**
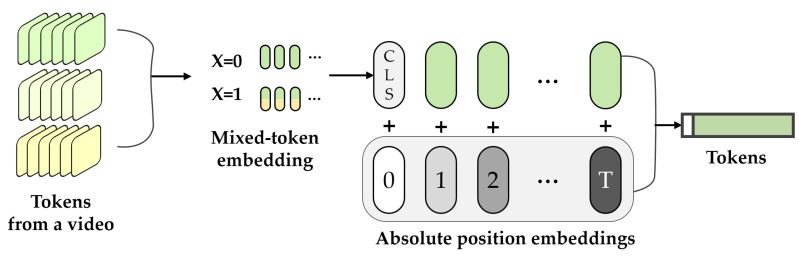
Mixed-token embedding method.

As shown in Figure 9, mixed-token embedding was performed before the transformer encoder. In addition, a learned classification embedding $z_{cls}$ was added to the token sequence generated from the video. $z_{cls}$ functioned as a final representation for classification at the final layer of the encoder. Thereafter, position embeddings *p* were added to maintain location information (Section 3.2.3).

(8) $$z=\left[z_{cls},\;z_{mix}\right]+p$$

#### 3.2.3. Positional Embeddings

**Spatial Positional Embedding**. Position embeddings are positional values that are added to input tokens to represent the positional information of each token that was missed in the process of tokenization in transformer-based models. The video transformer model [42] has $n_{t}$ times more tokens than the image transformer model [40]; thus, it needs $n_{t}$ times more position embeddings. Thus, p for the spatial transformer encoder initialized the learned location embeddings repeatedly in each layer $L_{S}$ as per time $n_{t}$, and then added them to the input token.

**Temporal Positional Encoding**. The spatial transformer encoder in SMaTE equally applied a learned position embedding of bidirectional encoder representations from transformers (BERT) [74], which also perform well in vision transformers [40,42]. This is because it also models spatial interactions with the same time index and there are no significant differences between the transformer encoder of ViT models [40].

Absolute position encoding functions efficiently; however, the number of temporal tokens that the model can process is limited. Moreover, it may not be fully utilized, because the temporal transformer encoder models interactions over time rather than dealing with highly structured data such as images. Thus, this study proposed two fixed-variant relative position encoding methods: temporal positional encoding (TPE) for position representations that are not restricted for long sequences and are appropriately generalized for time. TPE is defined as Equations (9) and (10):

(9) $$TPE_{1}\left(pos\right)=\frac{1}{1+e^{-\alpha \frac{2pos-L}{L}}}$$

(10) $$TPE_{2}\left(pos\right)=\frac{pos^{\alpha }}{L}$$

where position of the token $z_{t}$ is $pos\in \left[0,\;L-1\right]$ in the input sequence $z_{t}=\left(z_{0},\;\cdots ,\;z_{L-1}\right)$, and *L* is the maximum length of a token sequence output from the spatial transformer encoder. All values were normalized to *L* such that they were located between [0,1]. In contrast to conventional methods, TPE is a fixed encoding that represents a position value rather than an embedding with learnable parameters.

**Figure 10:**
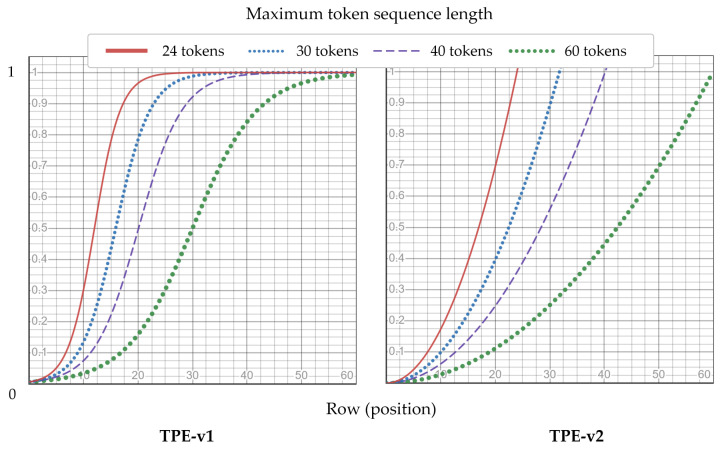
Temporal positional encoding (TPE)-v1 and TEP-v2 graphs showing the fixed position values according to the token position by the maximum length of the temporal token sequence.

Figure 10 shows the two relative position encoding methods for various temporal token lengths. Although the position encoding values varied depending on the token sequence length, SMaTE applied the same frame sampling to the video. Consequently, the input tokens possessed the same sequence length, and thus, even for different input data, each position encoding value was applied equally.

#### 3.2.4. Model Architecture

**Figure 11:**
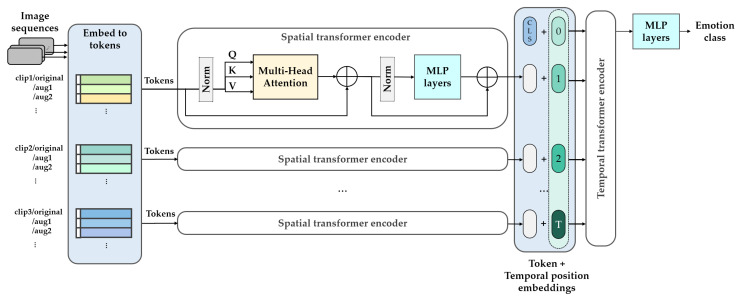
Illustration of the detailed model architecture in the SMaTE framework.

Figure 11 shows the structure of the SMaTE model based on the factored encoder–decoder of ViViT [42]. The transformer model in SMaTE comprised two transformer encoders: spatial transformer encoder (STE) and temporal transformer encoder (TTE). The transformer encoders were connected in series.

The STE received tokens extracted through a mixed-token embedding from preprocessed video, as described in Section 3.2.2. The STE modeled the spatial interaction between the input tokens, wherein each token was a mixed-token extracted from original and augmented clips each. In addition, all tokens were extracted from the same time index and different spatial indexes.

The TTE obtained frame-level representations $h_{i}$, which were concatenated by the application of the temporal position encoding. The representations $h_{i}$ is a group of output $z_{cls}$ for each time index of STE. The TTE modeled the interaction between different tokens of the time index and returned the final token $z_{cls}$ for classification from the last layer. Further, MLP head classified the emotion class through the $z_{cls}$ token. Thus, the attention was calculated separately in spatial and temporal dimensions, corresponding to “late fusion”, wherein the output was fused later in the network among approaches that fused information across time domains [75].

## 4. Results

First, the experimental setup and development environment are discussed in Section 4.1. Thereafter, the performance improvement for both datasets is presented in Section 4.2. The ViViT-Base (ViViT-B)/16 × 2/factorized encoder (FE) was employed as a backbone architecture (Section 3.2). Because ViViT-B [42] follows that of ViT [40] and BERT [74], the same setup was applied to the proposed model SMaTE in all experiments: the number of transformer layers *L* was 12, number of self-attention block $N_{H}$ was 12, and hidden size *d* was 768.

### 4.1. Experimental Setup

#### 4.1.1. Dataset

**CK+**. The CK+ dataset is facial expression sequence data photographed in a laboratory environment released for automatic facial expression recognition. Each image sequence is labeled as one of the seven expression classes (anger, contempt, disgust, fear, happiness, sadness, and surprise), showing a change in the face from a neutral expression to the target emotion. Further, all videos were shot at 30 fps with a resolution of 640 × 490 or 640 × 480. It is the most commonly used benchmark set for facial recognition evaluation because it contains a total of 593 image sequences for 123 experimental participants of different genders and ages ranging from 18 to 50.

**AFEW**. The AFEW dataset is a video emotion recognition dataset for automatic emotion recognition under various conditions in a wild environment. It was used as a training and benchmark database from 2013 to 2019 in EmotiW, the emotion recognition challenge, as part of the ACM International Conference on Multimodal Interaction. In addition, it is widely used as a wild FER benchmark set for video as well as in the challenge. AFEW was developed as a semiautomatic process using data from movies and reality TV, and each image sequence is labeled as one of seven emotion classes (anger, disgust, fear, happiness, neutral, sadness, and surprise). The subjects (actors) of 330 or more belong to a wide range of ages from 1 to 77. The videos have a variety of scenarios, including indoors, outdoors, at night, various different poses, and several people gathering. Further, most samples contain background noise much closer to the actual environment than laboratory control conditions. The entire dataset is divided into three sets: 773, 383, and 653 video clips in the training, validation set, and test sets, respectively, thus totaling 1809 videos with 113,355 total frames. As the test set has no labels disclosed, the performance is evaluated through the validation set. Moreover, it is an imbalanced dataset with significantly fewer samples of fear, disgust, and surprise emotions. This study extracted 130 samples from the validation set, balancing between classes, for use as a test set.

#### 4.1.2. Performance Metrics

In multiclass classification, top-N accuracy is the ratio of facial expression images in which the top-N predictions with high probability in the model result as correctly guessed for the actual emotion class. We use top-1 accuracy, in which one prediction with the highest probability matches the expected answer as a performance metric.

### 4.2. Ablation Study

Video clips were resized to a resolution of 256×256 and center and randoms crop of 224×224. For all experiments, videos were sampled into 25 frames with a stride of 2 on CK+ and a stride of 3 on AFEW to fit the same number of tokens. First, a backbone architecture for the CK+ and AFEW was evaluated, as presented in Table 1. The input was 25 frames, and tokens were generated as tubelet size t=2 using the tubelet embedding. ViViT exhibited low performance owing to small datasets (both CK+ and AFEW), with a larger accuracy drop on CK+, compared to CNN-based models performed in Section 2.

**Position information variants**. This study applied the proposed TPE to the temporal transformer encoder in the transformer model of the ViViT-B/16×2 FE pretrained on Kinetics400. Subsequently, the model learned to compare the effects of different position embedding methods on CK+ and AFEW. Table 2 indicates that the proposed simple fixed-position encoding method exhibited good performance, leading to higher accuracy than BERT’s learned position embedding baseline. TPE-v1 achieved a 43.18% accuracy on CK+ and 36.66% on AFEW. However, TPE-v2 improved the accuracy of BERT by 1.51% on CK+ and 4.45% on AFEW, and showed better performance than the TPE-v2 (0.75% on CK+ and 2.78 % on AFEW). Therefore, TPE-v2, which showed the highest accuracy on all datasets, was used for comparison with ViViT-B/16×2 FE/BERT (baseline) in all subsequent experiments.

**Progressive data augmentation**. We observe progressive improvements based on BERT in Table 3 and TPE-v2 in Table 4 results obtained in the above experiments, applying feature extraction and data augmentation to each data and tokenizing using mixed-token embedding. Overall, significant improvements were obtained in both BERT and TPE-v2 methods. In Table 3 and Table 4, to progressively show the effect of each method, one preprocessing method was added to each row. Each row contains all the methods listed above. LBP improves the performance substantially and shows a larger performance improvement, of 19.43 % on CK+ and 2.49 % on AFEW, in the TPE-v2-based model than the BERT-based model. Data augmentation, including crop, flip, rotate, and color jitter, demonstrates the best performance improvement among data processing methods with accuracy increases of 32.02% for BERT and 37.02 % for TPE-v2 on CK+ datasets. Mixed-token embedding was applied last because it requires augmented data. As shown in Table 3 and Table 4, mixed-token embedding achieved maximum accuracy across all datasets. Finally, we observe that ViViT-B can be further improved by applying data preprocessing for facial expression recognition, and we validated the superiority of the proposed SMaTE framework by obtaining the best results in the TPE-v2-based model.

**Computation across the number of tokens**. The SMaTE framework was implemented using the JAX/FLAX and Scenic libraries. Time complexity experiments were conducted on RTX A6000 using CK+ datasets sampled in 16 frames with a stride of 2. Table 5 shows computations, GFLOPs and training speeds across the number of tokens, to simulate the speed to learn quickly. Training speed is the speed performance in training steps per minute, and the larger the number, the better. In Table 5, FLOP increases linearly and the training speeds decrease according to the crop size.

### 4.3. Comparison with State-of-the-Art

We compare our method SMaTE framework with SOTA methods based on ablation studies. We use the TPE-v2-based SMaTE framework, which achieves the highest top-1 accuracy among the proposed methods. The performances of each SOTA method are the same as they are in the papers, and methods are limited to image-sequence-based models for fair comparison. We find that the proposed method provides a top-1 accuracy that outperforms the previous methods for both datasets. Table 6 shows that our framework based on TPE-v2 advances the results of CK to 99.19%. For AFEW in Table 6, Ref. [34] is the CNN-based model that won the challenge. However, the proposed method shows about 1.69% higher accuracy than image-sequence-based [34].

## 5. Discussion

This study proposes a transformer model for facial recognition focusing on image sequences in video-based emotion recognition. Previous work focused on vision transformer for video for a wide range of video classification; the proposed study focused on effective application of a video vision transformer model to facial expression recognition in video. The proposed framework improved performance on video emotion recognition datasets in laboratory and wild environments, compared to previously studied ViViT. The method to effectively reinforce a small FER dataset for application of a video-based vision transformer model to facial expression recognition tasks was shown. Moreover, additional performance improvements were shown through the application of position encoding to enhance time-relationship modeling. In the future work, studies can be conducted to improve computational complexity, and to develop more generally applicable transformer-based models with sufficient face datasets for real-world FER applications.

## Figures and Tables

**Table 1:** Top-1 accuracy of baseline using video vision transformer base (ViViT-B) on CK+ and AFEW.

Method	CK+	AFEW	FLOPs ($\times 10^{9}$)	Params ($\times 10^{6}$)
ViViT-B/16 × 2 FE	42.42	37.22	213.93	171.45

**Table 2:** Comparison of position information methods on CK+ and AFEW. We report the top-1 accuracy using ViViT-B/16 × 2 factorized encoder (FE) as backbone.

Initialization	CK+	AFEW
BERT	42.42	34.99
TPE-V1	43.18	36.66
TPE-V2	**43.93**	**39.44**

**Table 3:** Performance comparisons of various data preprocessing methods on CK+ and AFEW datasets in BERT-based SMaTE framework.

	CK+	AFEW
Original	42.42	34.99
LBP	54.95	37.22
Crop, flip, rotate, color jitter	91.97	38.33
Mixed-token embedding	**98.39**	**38.88**

**Table 4:** Performance comparisons of various data preprocessing methods on CK+ and AFEW datasets in temporal positional encoding (TPE)-v2-based SMaTE framework.

	CK+	AFEW
Original	43.93	37.22
LBP	63.35	39.71
Crop, flip, rotate, color jitter	95.38	40.83
Mixed-token embedding	**99.19**	**41.38**

**Table 5:** Evaluation of time complexity (FLOPs and training steps per minute) for the SMaTE framework on RTX A6000 using CK+ datasets.

Crop Size	64	128	224	320
GFLOPs	12.5	46.3	142.6	301.8
Training speeds	23.5	22.6	20.3	17.3

**Table 6:** Comparisons of top-1 accuracy on CK and AFEW datasets.

Method	CK	AFEW
DeepEmotion [76]	98	-
HMTL [77]	98.23	-
C3D [34]	-	39.69
SMaTE (TPE-v2)	99.19	41.38

## Data Availability

The Extended Cohn–Kanade dataset (CK+) that supports the findings of this study is available in the University of Pittsburgh at http://www.jeffcohn.net/resources (accessed on 19 February 2020) with the permission of Professor Jeffrey Cohn. The Acted Facial Expressions In The Wild (AFEW) dataset that supports the findings of this study is available from the Emotion Recognition in the Wild Challenge (EmotiW) Organizers at https://sites.google.com/view/emotiw2020 (accessed on 2 January 2022) with the permission of Professor Abhinav Dhall.

## Abbreviations

The following abbreviations are used in this manuscript:

AFEW	Acted Facial Expressions in the Wild
BERT	Bidirectional encoder representations from transformers
CK+	Extended Cohn–Kanade
CNN	Convolutional neural networks
FE	Factorized encoder
FER	Facial expression recognition
HCI	Human–computer interactions
LBP	Local binary patterns
RNN	Recurrent neural networks
SER	Speech emotion recognition
SMaTE	Segment-level feature mixing and temporal encoding
STE	Spatial transformer encoder
TPE	Temporal positional encoding
TTE	Temporal transformer encoder
ViT	Vision transformer
ViViT	Video vision transformer
